# Development and validation of pyroptosis-related lncRNAs prediction model for bladder cancer

**DOI:** 10.1042/BSR20212253

**Published:** 2022-01-28

**Authors:** Thongher Lia, Yanxiang Shao, Parbatraj Regmi, Xiang Li

**Affiliations:** 1 Department of Urology, West China Hospital, Sichuan University, Chengdu 610041, Sichuan Province, China; 2 Department of Biliary Surgery, West China Hospital, Sichuan University, Chengdu 610041, Sichuan Province, China

**Keywords:** bladder cancer, long non-coding RNA, prognostic biomarker, pyroptosis

## Abstract

Bladder cancer (BLCA) is one of the highly heterogeneous disorders accompanied by a poor prognosis. The present study aimed to construct a model based on pyroptosis-related long-stranded non-coding RNA (lncRNA) to evaluate the potential prognostic application in bladder cancer. The mRNA expression profiles of bladder cancer patients and corresponding clinical data were downloaded from the public database from The Cancer Genome Atlas (TCGA). Pyroptosis-related lncRNAs were identified by utilizing a co-expression network of pyroptosis-related genes and lncRNAs. The lncRNA was further screened by univariate Cox regression analysis. Finally, eight pyroptosis-related lncRNA markers were established using least absolute shrinkage and selection operator (Lasso) regression and multivariate Cox regression analyses. Patients were separated into high- and low-risk groups based on the performance value of the median risk score. Patients in the high-risk group had significantly poorer overall survival (OS) than those in the low-risk group (*P*<0.001). In multivariate Cox regression analysis, the risk score was an independent predictive factor of OS (HR > 1, *P*<0.01). The areas under the curve (AUCs) of the 3- and 5-year OS in the receiver operating characteristic (ROC) curve were 0.742 and 0.739, respectively. In conclusion, these eight pyroptosis-related lncRNA and their markers may be potential molecular markers and therapeutic targets for bladder cancer patients.

## Introduction

Bladder cancer (BLCA) is caused by malignant tumors that grow on the mucosa of the bladder and ranks tenth among systemic malignancies worldwide [1]. With the influence of factors such as aging, environmental pollution and smoking, the morbidity and mortality of bladder cancer has been increasing [2]. Bladder cancer that has not invaded to the deeper layers of the bladder wall are treated with transurethral resection of the tumor and total cystectomy is preserved for patients with tumor invasion into the muscular layer of the bladder [3]. Over the past few decades, despite improvements in surgical and non-surgical treatments for bladder cancer, bladder cancer still has a high risk of recurrence and is associated with a poor prognosis [4–6]. There are currently no effective therapeutic targets for bladder cancer in clinical medicine [7].

Non-coding RNA (ncRNA), including microRNA (miRNA), long-stranded non-coding RNA (lncRNA), and circular RNA (circRNA) play important roles in cell growth [8]. With the advancement of transcriptome analysis, the role of dysfunctional lncRNAs in cancer has attracted considerable attention [9–11]. LncRNAs are ncRNAs ranging from 200 to 100000 nucleotides in length [12]. There has been growing evidence that aberrant lncRNA expression is associated with tumor development, diagnosis, and prognosis with the development of tumors [13,14].

Pyroptosis is a programmed form of cell death caused by an inflammatory response [15]. Not only it does play an important role in infectious diseases, but is also associated with cardiovascular diseases, central nervous system diseases, and tumors [15–17]. Pyroptosis is involved in tumorigenesis, growth, and metastasis [18]. Pyroptosis, on the one hand, stalls the growth of tumor cells and exhibits anti-tumor activity [19,20]. On the other hand, an inflammatory component is produced during scorch death, which activates pro-inflammatory cytokines that provide a microenvironment for tumor cell growth and contribute to tumor growth [21]. However, the association of the expression of pyroptosis-related lncRNA with bladder tumor developments and prognosis remains to be investigated in great detail.

In the present study, we performed an analysis of lncRNA expression datasets from The Cancer Genome Atlas (TCGA) for bladder cancer and screened for pyroptosis-related lncRNAs of prognostic value. We identified eight pyroptosis-related lncRNA signatures that have the potential to predict survival prognosis in bladder cancer patients.

## Materials and methods

### Data source

TCGA datasets on bladder cancer and corresponding clinical characteristics of patients were downloaded from the UCSC Xane website (UCSC Xena (xenabrowser.net)) which included 414 BLCA samples and 19 normal tissues. We incorporated patients who had clinical data with a follow-up time of more than 30 days.

### Curtaining data for lncRNAs and pyroptosis-related genes

Bladder cancer data were annotated by Gencode (GENCODE version GRCh38) GTF file in the present study. Normalization of RNA-seq data expression profiles was performed by using log2 transformations. The extraction of mRNA and lncRNA genes from RNA-seq data with the Perl software (version: Strawberry-Perl-5.32.1). Pyroptosis-related genes were obtained from the prior review [22,23]. The Pearson correlation was used to measure the correlation between lncRNAs and pyroptosis-related genes. Pyroptosis-related lncRNAs were determined as those with a Pearson correlation coefficient of 0.4 and a *P*-value of <0.001. The Pearson correlation was used to measure the correlation between lncRNAs and pyroptosis-related genes. Pyroptosis-related lncRNAs were determined as those with a Pearson correlation coefficient of 0.4 and a *P*-value of <0.001.

### Construction of the pyroptosis-related prognostic lncRNAs

Univariate Cox regression was used to analyze the prognostic value of pyroptosis-related lncRNAs. The least absolute shrinkage and selection operator (Lasso) regression was used to incorporate the pyroptosis-related lncRNAs with *P*-value <0.01 in the univariate analysis. Eventually, to create a risk score, the Lasso regression results were incorporated into a multivariate Cox model. The risk signature was constructed by multiplying linear combination of the pyroptosis-related lncRNA expression levels with a regression coefficient (β) (risk score = expression level of Gene1 ∗ β1 + expression level of Gene2 ∗ β2 +…+ expression level of Gene(n) ∗ β(n)). The patients were classified into high- and low-risk groups based on the median risk score. A log-rank test was used to compare the survival differences between the two groups.

### Constructing a prognostic model

The nomogram was designed to predict the patient’s survival. The model’s accuracy was assessed using the C- index, calibration curve, and linear regression (receiver operating characteristic, ROC) curve. To see if risk score was an independent predictor of prognosis, we utilized multivariate Cox regression analysis including clinical characteristics.

### Immune infiltration analysis

CIBERSORT and ESTIMATE were used to estimate the immune cell content of each bladder cancer patient, according to the abundance of immune cells between the high- and low-risk groups. Immune-related molecules were further analyzed to obtain information on the immune infiltration of bladder cancer.

### Enrichment analyses of pyroptosis-related genes

Gene set enrichement analysis (GSEA_4.1.0 version) was performed to explain the function of gene expression data enrichment. Gene ontologies (GOs) enrichment and Kyoto Encyclopedia of Genes and Genomes (KEGG) pathway analyses were performed. We have explored the functional enrichment of prognostic lncRNAs that are associated with pyroptosis-related pathways based on the high- and low-risk groups.

### Statistical analysis

The Kaplan–Meier process was then used to establish survival curves, which were then compared using the Log-rank test. The prognostic implications of pyroptosis-related lncRNA features and clinicopathological data were explored using Cox proportional hazards regression model, Lasso regression, and proportional hazards assumption for a Cox regression model for statistical analysis, the R programming language (version 4.1) was utilized. Statistical tests were conducted in both directions, with a *P*-value <0.01 deemed statistical significance.

## Results

### Construction of a co-expression network

Throughout the TCGA-BLCA data, 14142 lncRNAs were identified. A total of 259 pyroptosis-related genes were identified, of which 33 were expressed as mRNAs in bladder cancer. To investigate pyroptosis-related lncRNAs, a pyroptosis-related gene lncRNA co-expression network was developed. Finally, 1025 pyroptosis-related lncRNAs were identified.

### Identification of prognostic pyroptosis-related lncRNA signature

**Figure 1:**
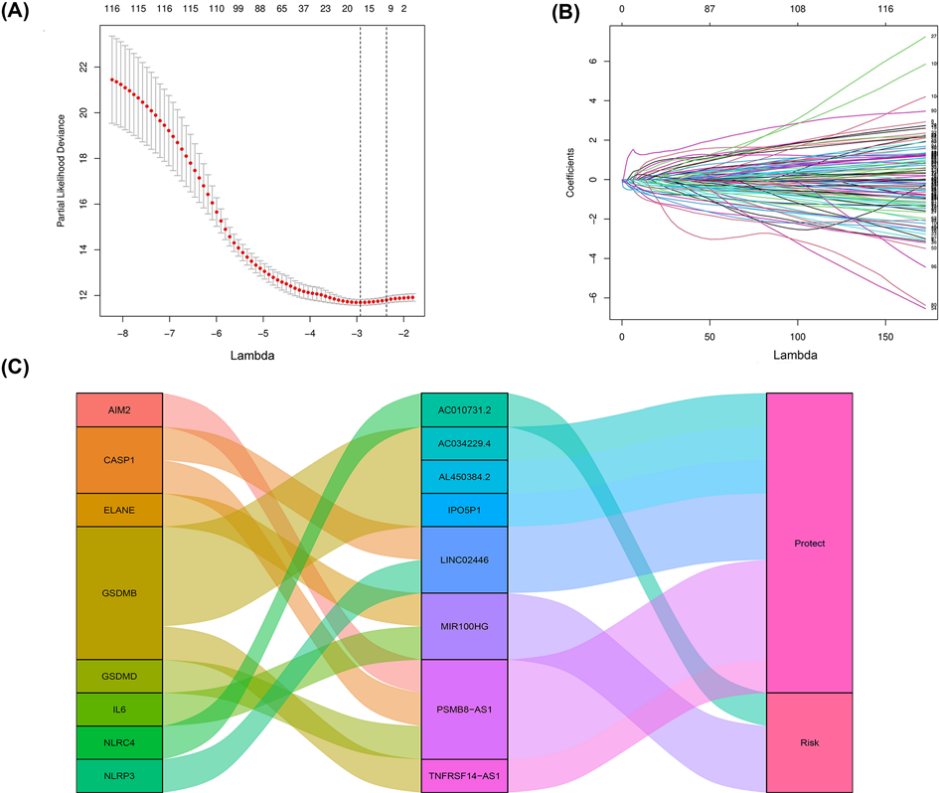
Identification of a 16 pyroptosis-related lncRNA risk signature for overall survival by Lasso regression analysis in TCGA cohort (**A**) Cross-validation for tuning parameter selection in the proportional hazards model. (**B**) Lasso coefficient spectrum of 16 pyroptosis-related lncRNAs in bladder cancer. (**C**) Sankey diagram showed the association among prognostic pyroptosis-related lncRNAs, pyroptosis-related mRNAs, and risk types.

**Figure 2:**
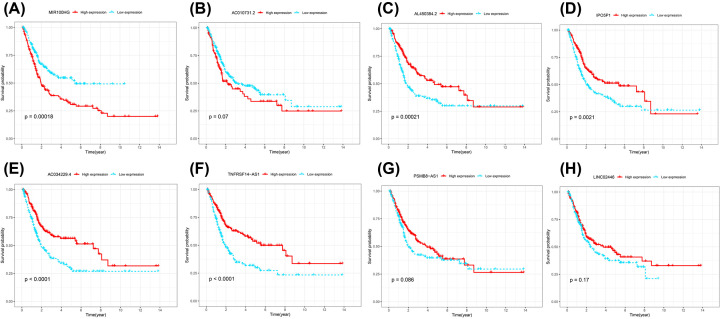
Kaplan–Meier survival analysis of eight pyroptosis-related lncRNAs (**A**) MIR100HG, (**B**) AC010731.2, (**C**) AL450384.2, (**D**) IPO5P1, (**E**) AC034229.4, (**F**) TNFRSF14-AS1, (**G**) PSMB8-AS1, (**H**) LINC02446 associated with prognosis in patients with bladder cancer.

Based on the results of univariate Cox analysis, 119 pyroptosis-related lncRNAs showed a predictive value for bladder cancer patients (*P*<0.01, Supplementary Table S1). Through Lasso regression, the 16 pyroptosis-related lncRNAs were identified (Figure 1A,B and Supplementary Table S2). In the multivariate analysis, eight lncRNAs were found to be associated with prognostic factors in patients (Figure 1C and Table 1). Of these, two lncRNAs (MIR100HG, AC010731.2) were the poor prognostic factors for patients (Figure 2A,B) and six lncRNAs (AL450384.2, IPO5P1, AC034229.4, TNFRSF14-AS1, PSMB8-AS1, LINC02446) were the favorable prognostic factors for patients (Figure 2C–H). These eight lncRNAs were utilized as signature lncRNAs related to pyroptosis. The formula of the risk score was as follows: Risk score = (1.113718024*AC010731.2) +(0.244249497*MIR100HG) + (−0.784173266*AC034229.4) + (−0.359894228*AL450384.2) +(−0.225050249*IPO5P1) + (−0.40405957*LINC02446) + (−0.289612092*PSMB8-AS1) + (−0.5224276 *TNFRSF14-AS1).

**Table 1:** Multivariate analysis of the pyroptosis-related lncRNAs cohort

LncRNA	Coefficient	HR	HR.95L	HR.95H	*P*-value
AC010731.2	1.113718	3.045661	1.821714	5.091936	2.16E-05
AC034229.4	−0.78417	0.456497	0.194484	1.071501	0.071652
AL450384.2	−0.35989	0.69775	0.480704	1.012796	0.058347
IPO5P1	−0.22505	0.798476	0.62583	1.01875	0.070216
LINC02446	−0.40406	0.667604	0.495533	0.899426	0.007885
MIR100HG	0.244249	1.276663	0.997228	1.634399	0.052631
PSMB8-AS1	−0.28961	0.748554	0.578261	0.968997	0.02787
TNFRSF14-AS1	−0.52243	0.593079	0.378479	0.929359	0.022629

### The prognostic impact of the established signatures

**Figure 3:**
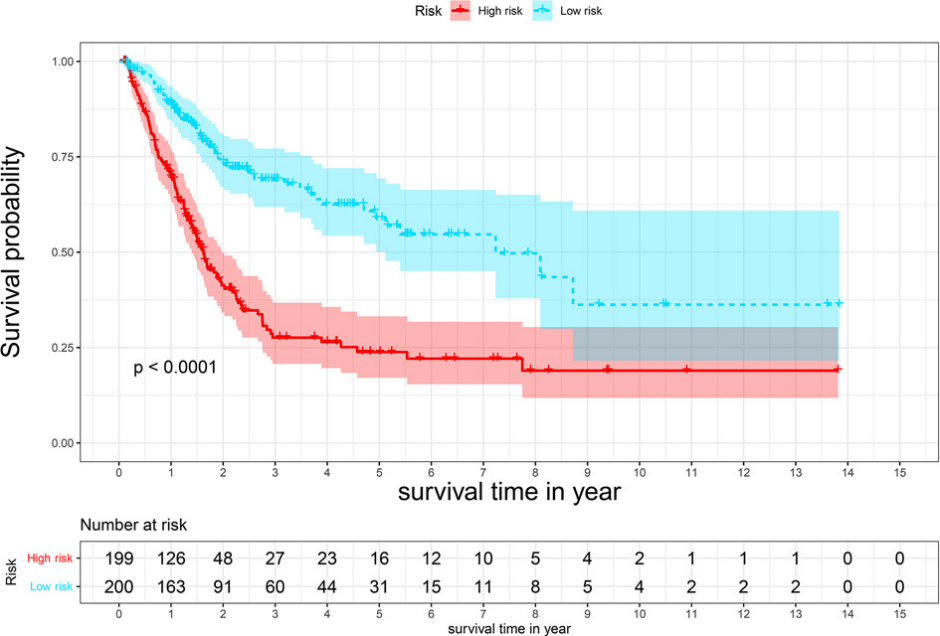
The risk score of Kaplan–Meier survival curves based on eight pyroptosis-related lncRNAs

**Figure 4:**
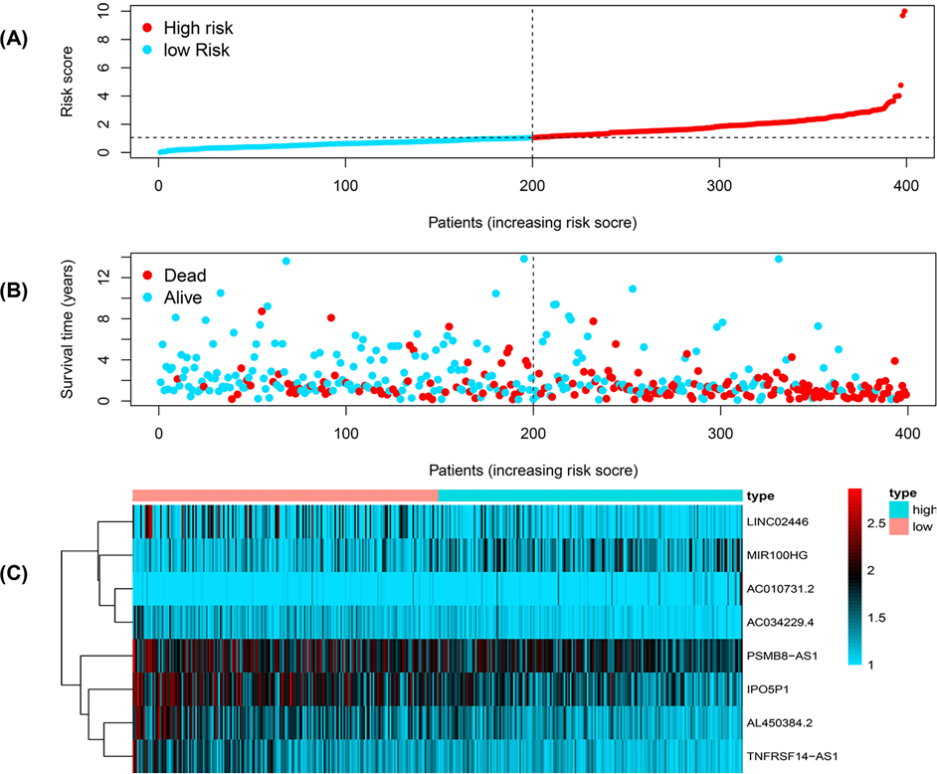
The analysis distribution of survival status and heat map in pyroptosis-related lncRNA signature for patients with bladder cancer (**A**) Distribution of risk score status of bladder cancer. (**B**) The survival time of the patient. (**C**) Heat map of pyroptosis-related lncRNA expression profiles in the prognostic signature of bladder cancer.

We calculated risk scores for each patient in the test dataset and divided patients into low- and high-risk groups based on the median risk score. Patients in the low-risk group overall survival (OS) had significantly improved outcomes (*P*<0.001) (Figure 3). The risk score had a significant influence on the prognosis of individuals with bladder cancer according to Cox regression analysis (Figure 4).

### The pyroptosis-related lncRNA signature’s clinical importance

**Figure 5:**
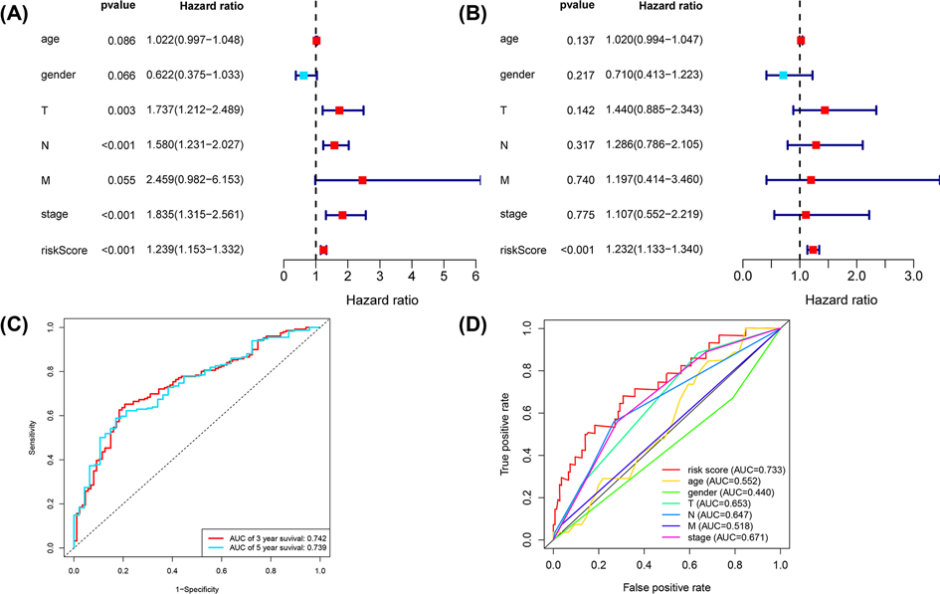
Univariate and multivariate Cox analyses of prognostic pyroptosis-related lncRNA risk scores for bladder cancer (**A**) Result of univariate Cox regression. (**B**) Result of multivariate Cox regression. (**C**) ROC curve analysis shows the prognostic prediction of 3 and 5 years. (**D**) Prognostic value of composite nomogram based on several prognostic factors.

**Figure 6:**
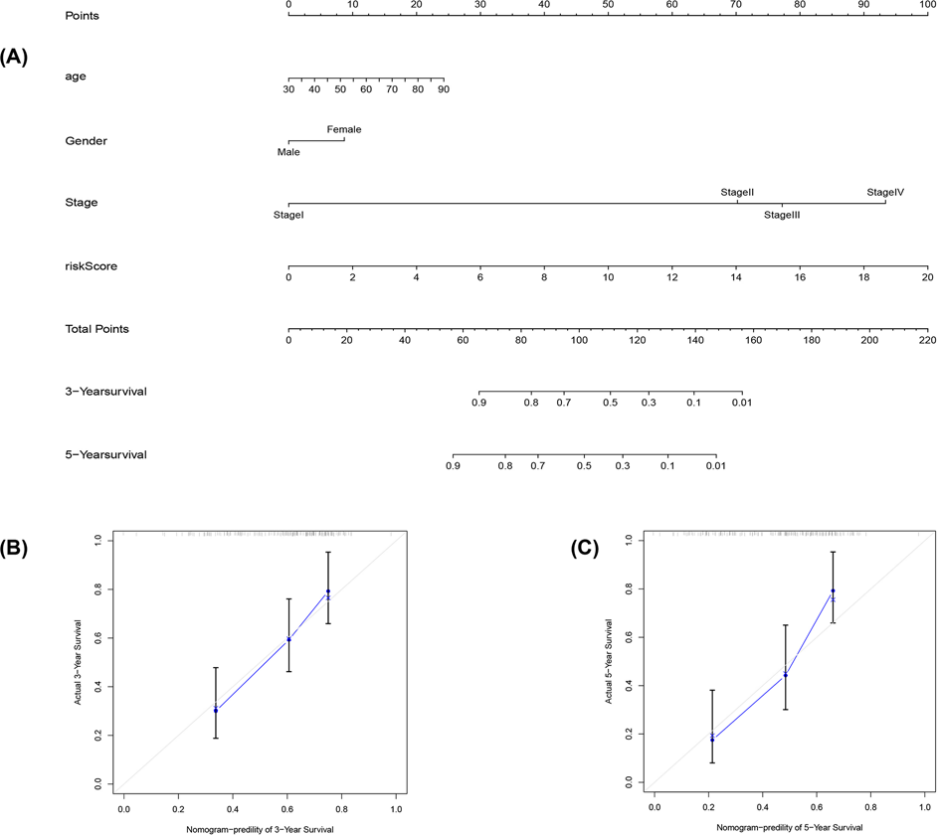
Prognostic model evaluation of eight pyroptosis-related associated lncRNAs (**A**) Nomogram predicting 3- and 5-year OS based on age, gender, stage, and risk score. (**B**) Nomogram-predicted probability of 3-year survival. (**C**) Nomogram-predicted probability of 5-year survival (the 45º dotted line represents a perfect prediction, and the blue lines represent the predictive performance of the nomogram).

The risk score (HR = 1.239; 95% CI: 1.153–1.332; *P*<0.001) and N-stage (HR: 1.580; 95% CI: 1.231–2.027; *P*<0.001) were independent prognostic predictors in a univariate Cox regression analysis (Table 2, Figure 5A). In multivariate analysis, only the risk score (HR = 1.232; 95% CI = 1.133−1.340; *P*<0.001, Table 2, Figure 5B) was a significant prognostic predictor. Further, the area under the curve (AUC) values for areas under the ROC curve predicting 3- and 5-year survivals were 0.742 and 0.739, respectively (Figure 5C). The AUC for areas under the multivariate ROC curve predicting showed definite predictive modeling ability, mainly through risk score, T-stage, and N-stage were 0.733, 0.653, and 0.647, respectively (Figure 5D). We validated the nomograms by testing the proportional hazards assumption of the Cox regression model to the prediction ability of the assumption model (Supplementary Figure S1). There was no statistical significance for each covariable (*P*>0.05) and no statistical significance for the global test (Supplementary Table S3). For each covariate, we generated the correlations of the corresponding sets of standardized Schoenfeld residuals with time to test for independence between residuals and time (Supplementary Figure S1), the results showed that the linear relationship between residuals and time is not significant in the proportional risk hypothesis. In the Nomogram plots, we predicted the OS of bladder cancer patients at 3 and 5 years by the association of risk score and stage, as shown in (Figure 6A–C). In the prognosis model of bladder cancer, the C-index was 0.711.

**Table 2:** Univariate and multivariate Cox regression analyses were used to assess the clinical features and risk scores of bladder cancer

Variable	Univariate Cox regression				Multivariate Cox regression			
	HR	HR.95L	HR.95H	*P*-value	HR	HR.95L	HR.95H	*P*-value
Age	1.022028	0.996921	1.047767	0.085988	1.018527	0.992694	1.045032	0.161348
Gender	0.621914	0.374543	1.032664	0.066397	0.600981	0.356789	1.012305	0.055621
T	1.736894	1.21213	2.488843	0.002628	1.294533	0.795159	2.107522	0.299192
N	1.579951	1.231276	2.027365	0.000324	1.099677	0.67681	1.78675	0.701219
M	2.458538	0.982327	6.153155	0.054622	1.604976	0.557005	4.624646	0.38092
Stage	1.835178	1.314974	2.561174	0.000357	1.36429	0.699753	2.659918	0.361829
Risk score	1.047395	1.028431	1.066709	6.80E-07	1.041398	1.020574	1.062647	8.28E-05

### Correlation of the expression of the eight pyroptosis-related lncRNAs with clinicopathological factors

Further, we applied whether eight pyroptosis-related associated lncRNAs are involved in the development of bladder cancer, and we investigated the relationship between the expression of eight pyroptosis-related lncRNAs and clinicopathological factors. Of the eight lncRNAs associated with pyroptosis, the results showed that AL450384.2, MIR100HG, IPO5P1, and TNFRSF14-AS1 were significantly associated with N_stage. AL450384.2, IPO5P1, LINC02446, and TNFRSF14-AS1 were significantly associated with T_stage, PSMB8-AS1 was significantly associated with M_stage, and IPO5P1 was significantly associated with gender, respectively (Supplementary Figure S2).

### Relationship between pyroptosis-related lncRNAs signature and immune cells infiltration

**Figure 7:**
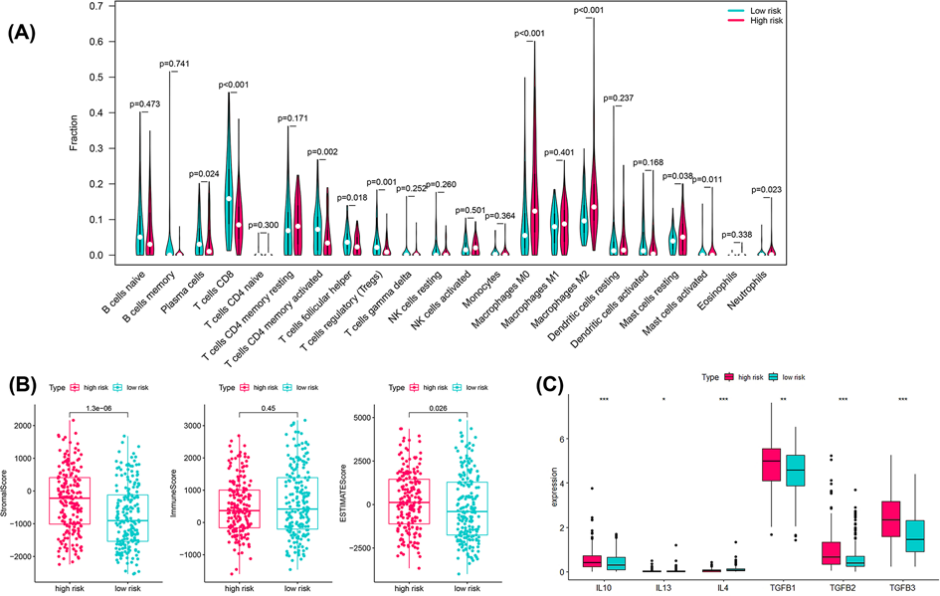
Immune cells infiltration analysis (**A**) The vioplots showed that 22 immune cells content in the high- and low-risk groups. *P<0.05; **P<0.01; ***P<0.001; ns, not significant. (**B**) ESTIMATE comparison of stromal, immune and tumor purity scores in high- and low-risk groups. (**C**) Expression of the immune suppressive cytokines between high- and low-risk groups. *P<0.05, **P<0.01, ***P<0.001.

We evaluated the infiltration of the 22 types of immune cells in the TCGA database by the CIBERSORT algorithm estimation and found that 10 types of immune cells were significantly different between the high- and low-risk groups (*P*<0.05) (Figure 7A). These included plasma cells, T cells CD8, T cells CD4 memory activated, T cells follicular helper, T cells regulatory (Tregs), Macrophages M0, Macrophages M2, Mast cells resting, Mast cells activated, and Neutrophils. Figure 7B shows the ESTIMATE scores and the differences between the low- and high-risk groups in the tumor microenvironment, stromal score, and immune score. The stromal score was significantly higher in the high-risk group (*P*<0.001), whereas the immune scores were not statistically significant in the high- and low-risk groups. Moreover, the combined ESTIMATE scores were higher in the high-risk group than in the low-risk group (*P*<0.05).

### High-risk tumors show an immunosuppressive phenotype

Our results from CIBERSORT calculations revealed that macrophages were significantly up-regulated in all high-risk groups, suggesting that an immunosuppressive phenotype may exist in these tumors. Most of the chemokines (IL10, IL13, IL4 TGFB1, TGFB2, TGFB3) involved in immunosuppressive processes through macrophage induction and were also significantly up-regulated in the high-risk group (*P*<0.05) (Figure 7C). These data suggest that high-risk patients exhibit inertia in anti-tumor immunity, which could potentially contribute to their poor prognosis.

### Functional analysis

**Figure 8:**
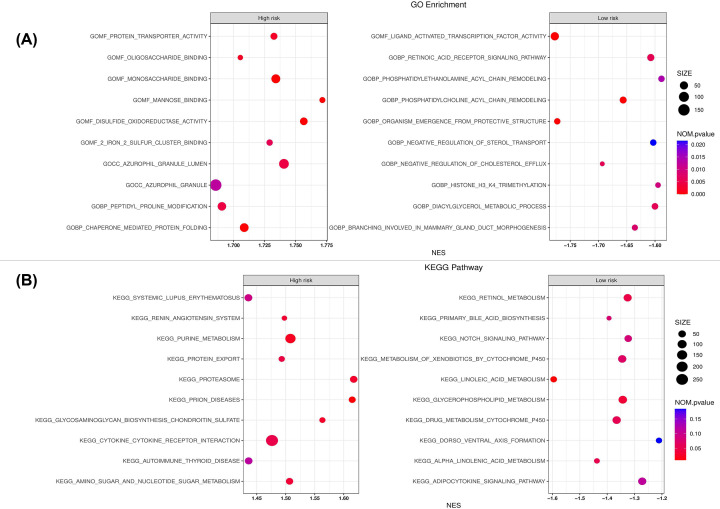
GO and KEGG enrichment analyses of pyroptosis-related lncRNAs (**A**) GO enrichment analysis of top ten high-risk groups (on left) and top ten low-risk groups (on right) of pyroptosis-related lncRNAs. (**B**) Top ten KEGG pathways in high-risk groups (on left) and top ten KEGG pathways in low-risk groups (on right) of pyroptosis-related lncRNAs.

We performed GO enrichment and KEGG pathways analysis of patients’ samples by high-risk score. GO enrichment shows that the top ten of functions of pyroptosis-related lncRNAs in high-risk groups were mostly focused on protein transporter activity, oligosaccharide binding, monosaccharide binding, mannose-binding, disulfide oxidoreductase binding, two iron–two sulfur cluster binding, azurophil granule lumen, azurophil granule, peptidyl proline modification, chaperone-mediated protein folding (Figure 8A and Supplementary Table S4). Whereas the top ten functions of the low-risk groups were ligand-activated transcription factor activity, organism emergence from protective structure, negative regulation of cholesterol efflux, phosphatidylcholine acyl chain remodeling, branching involved in mammary gland duct morphogenesis, retinoic acid receptor signaling pathway, negative regulation of sterol transport, diacylglycerol metabolic process, histone H3K4 trimethylation, phosphatidylethanolamine acyl chain remodeling (Figure 8A and Supplementary Table S5). GSEA showed the KEGG pathways analysis of pyroptosis-related lncRNAs in high-risk groups significantly enriched in the amino sugar and nucleotide sugar metabolism, autoimmune thyroid disease, cytokine receptor interaction, glycosaminoglycan biosynthesis chondroitin sulfate, prion diseases, proteasome, protein export, purine metabolism, renin–angiotensin system, systemic lupus erythematosus (Figure 8B and Supplementary Table S6). Interestingly, the KEGG pathway of pyroptosis-related lncRNAs in low-risk groups was mainly involved in metabolic pathways and adipocytokine signaling pathway, Notch signaling pathway, dorsoventral axis formation, primary bile acid biosynthesis (Figure 8B and Supplementary Table S7).

## Discussion

At present, radical cystectomy combined with pelvic lymph node dissection and chemotherapy is the best treatment for muscle-invasive bladder cancer [3]. However, approximately 50% of patients die from metastatic disease [24], and the prognosis for patients with advanced and metastatic bladder cancer has remained unsatisfactory. Therefore, the prognostic role of molecular biomarkers is particularly important and may improve the prognosis of patients with this disorder [25]. The currently available studies showed that lncRNAs play a crucial regulatory role in pyroptosis-related biological processes in various cancer types [26]. However, there are only a few methods for predicting pyroptosis-related lncRNAs in bladder cancer patients. Our results identified eight pyroptosis-related lncRNAs, out of which two lncRNAs (AC010731.2 and MIR100HG) were associated with a poor prognosis of bladder cancer.

PSMB8-AS1 is found to have been associated with a variety of tumors. PSMB8-AS1 improves the proliferation and metastasis of PC cells by sponging miR-382-3p to up-regulate STAT1 expression. [27]. PSMB8-AS1 activated by ELK1 promotes cell proliferation in glioma via regulating miR-574-5p/RAB10 [28]. A study of epithelial–mesenchymal transition-related lncRNA signature correlating with the prognosis and progression in patients with bladder cancer suggests that PSMB8-AS1 and AC073534.1 could be used to predict the prognosis and progression of bladder cancer patients [29]. LINCOA2246 is a genomic instability-related lncRNA for bladder cancer, which may be of prognostic value and guide the clinical management of bladder cancer patients [30]. LINC02446 may influence the proliferation, migration, and invasion of bladder cancer cells. In addition, it was found to bind EIF3G protein and regulate the stability of EIF3G protein, which in turn inhibited the mTOR signaling pathway [31]. It also stimulates melanoma progression by reducing tumor-protecting miR-891a-5p and miR-203b-3p. An article on immune gene-related lncRNA found that AL450384.2 may be an important indicator of prognosis in patients with bladder cancer [32]. A recent study on the prognosis of immune gene-related lncRNAs and immunotherapy suggests that IPO5P1 may be a prognostically important value in bladder cancer patients as well as a predictor of the efficacy of immunotherapy [33]. MIR100HG plays an important role in human tumors and is an lncRNA that has received a lot of attention in the tumors, and it is expressed differently in tumor tissues dependent on the type of tumor. MIR100HG is involved in tumor proliferation, migration, and invasion in breast cancer [34], liver cancer [35], and laryngeal squamous cell carcinoma [36]. Down-regulation of MIR100HG expression in gastric cancer inhibits proliferation, migration, and invasion of gastric cancer cells [37]. It has been reported that down-regulation of MIR100HG in acute megakaryocytic leukemia may lead to inhibition of M-07e cell proliferation and induction of apoptosis and necrosis through up-regulation of TGFb expression [38]. TNFRSF14-AS1 may be a prognostically associated marker for bladder cancer immunogene-related lncRNAs [32]. For AC010731.2 and AC034229.4, the prognostic role of lncRNAs in cancer has not been reported so far. Therefore, there is a need to further research how they affect the prognosis of bladder cancer patients through their pyroptosis effects.

Immunotherapy is emerging as a new treatment option for cancer, also in uroepithelial carcinoma, where immune cell infiltration and immune checkpoints in the tumor tissue play a role in promoting or inhibiting cancer cell proliferation, invasion, and migration [39,40]. To investigate the association between scorched lncRNA signaling and immune cell infiltration, we compared the contents of immune cell content groups with different risk scores and found that Macrophages M0, Macrophages M2, Mast cells resting, Mast cells activated, Neutrophils were significantly higher than those in the low-risk group, while Plasma cells, T cells CD8, T cells CD4 memory activated, T cells follicular helper, Tregs were higher in the low-risk group than in the high-risk group. Macrophages play an important role in tumor progression [41], and these macrophages, also known as tumor-associated macrophages (TAMs), can promote tumor cell growth through a variety of mechanisms, including enhanced angiogenesis, chemoresistance, and suppression of antitumor immunity [42]. Macrophages M2 ay primarily promote tumor cell genesis and metastasis, inhibit T cell-mediated antitumor immune responses, promote tumor angiogenesis, and lead to tumor progression [43]. In the development of tumors multiple stimuli, such as anti-tumor antibodies, hypoxia, cytokines and chemokines, can activate mast cells and their mediators in the tumor microenvironment, causing them to play important immunomodulatory roles in tumor promotion and anti-tumorigenesis [44]. There is growing research evidence that tumor-infiltrating neutrophils play an important role in tumor promotion, progression, and treatment resistance [45,46]. A study of tumor-infiltrating neutrophils predicting the benefit of adjuvant chemotherapy in patients with muscle-infiltrating bladder cancer suggests that tumor-infiltrating neutrophils can be an independent prognostic factor. High tumor-infiltrating neutrophils are associated with immunosuppression in MIBC environments [47]. Most interesting are the findings that Tregs significantly infiltrate the tumor microenvironment in patients with low-risk scores. The potential relationship between pyroptosis-related and Tregs in bladder cancer is unclear. In this study, we used the CIBERSOR calculation to infer immune cell infiltration in bladder cancer, and TAMs were up-regulated in the high-risk group. We further confirmed that cytokines involved in the immunosuppressive process (IL-4, IL-10, IL-13, TGF-β) were up-regulated in the high-risk group. Our findings show the great potential of our markers in predicting bladder cancer, which may be beneficial for immunotherapy targets in bladder malignancy.

Our study of eight markers of lncRNAs associated with pyroptosis-related significantly predicted the prognosis of bladder cancer patients. OS was longer in the low-risk group than in the high-risk group. Three- and five-year survival corresponded to an area under the ROC curve of 0.773 and 0.767, respectively. Our findings indicated that the risk score feature may help predict survival and thus may be considered as an important prognostic predictor. The model showed modest predictive performance and dependability, according to the findings of the C-index, ROC curve, and Calibration curve. The eight lncRNAs associated with pyroptosis-related were significantly correlated with stage and gender, suggesting that the eight lncRNAs associated with pyroptosis-related may vary in the extent of bladder cancer, which could help doctors plan treatment accordingly and understand the prognosis and outcome of the disease.

However, our study has some limitations. We applied public databases to construct prognostic risk models for eight pyroptosis-related lncRNA through R language and statistical analysis. While these methods have already been applied and proven in many studies, to demonstrate the eight lncRNA associated with pyroptosis-related we need to conduct an in-depth study, which includes their function and molecular mechanisms.

## Conclusion

In summary, we have developed a prediction model based on eight pyroptosis-related lncRNAs (AC010731.2, MIR100HG, AC034229.4, AL450384.2, IPO5P1, LINC02446, PSMB8-AS1, TNFRSF14-AS1). This model may provide a new research strategy for exploring the pathogenesis of pyroptosis-related disorders and provide an individualized prediction of the prognosis of bladder cancer. Functional assessment and bioinformatics analysis revealed significant correlations in functionally enriched phases and pathways associated mainly with metabolic signaling pathways. Further studies are required to elucidate the mechanism of action of these pyroptosis-related lncRNAs in bladder cancer.

## Supplementary Material

Supplementary Figures S1-S2 and Supplementary Tables S1-S7

## Data Availability

All data generated or analyzed during the present study are included in the published article.

## Abbreviations

AUC: area under the curve
BLCA: bladder cancer
CI: Confidence Interval
C-index: Concordance-index
GO: gene ontology
GSEA: gene set enrichement analysis
HR: Hazard Ratio
KEGG: Kyoto Encyclopedia of Genes and Genomes
Lasso: least absolute shrinkage and selection operator
lncRNA: long non-coding RNA
OS: overall survival
PC: Pancreatic Cancer
ROC: receiver operating characteristic
TAM: tumor-associated macrophage
TCGA: The Cancer Genome Atlas
Treg: T cells regulatory
