# Supplementary material for: Development and validation of pyroptosis-related lncRNAs prediction model for bladder cancer
Source: Biosci Rep. 2022 Jan 28;42(1):BSR20212253. doi: 10.1042/BSR20212253 (PMC8799921; doi:10.1042/BSR20212253)
Supplement: Supplementary Figures S1-S2 and Supplementary Tables S1-S7 [file BSR-2021-2253_supp.pdf]

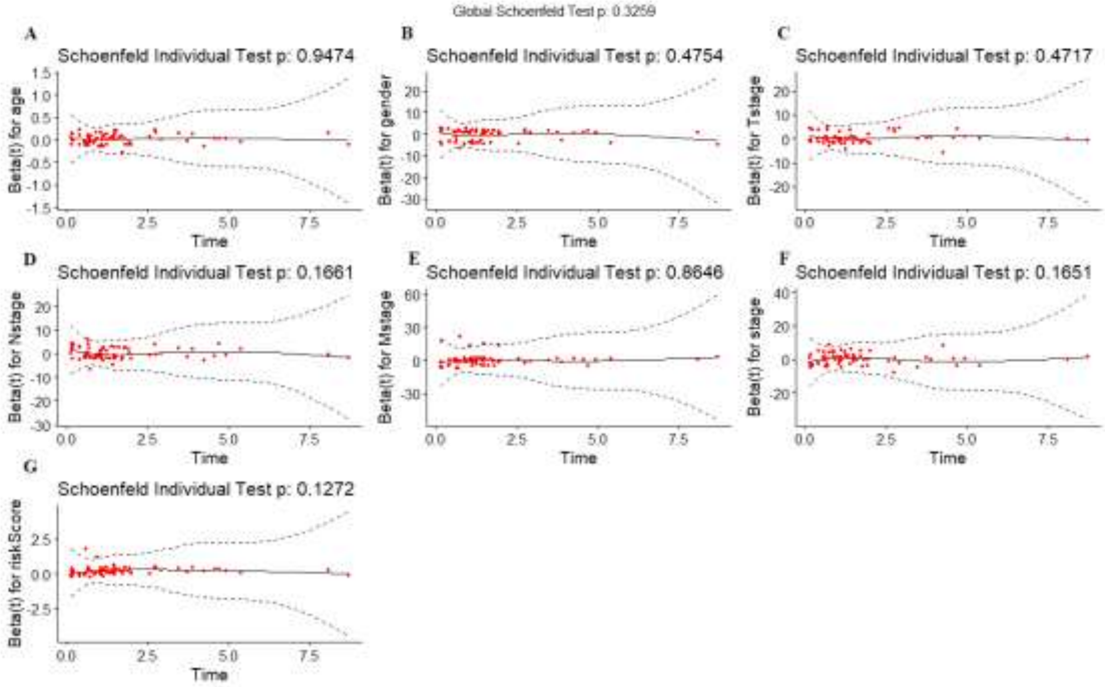

Figure S1: Correlation of each covariate generating standardized Schoenfeld residuals relative to time. (A) age, (B) gender, (C) T-stage, (D) N-stage, (E) M-stage, (F) stage, (G) risk score.

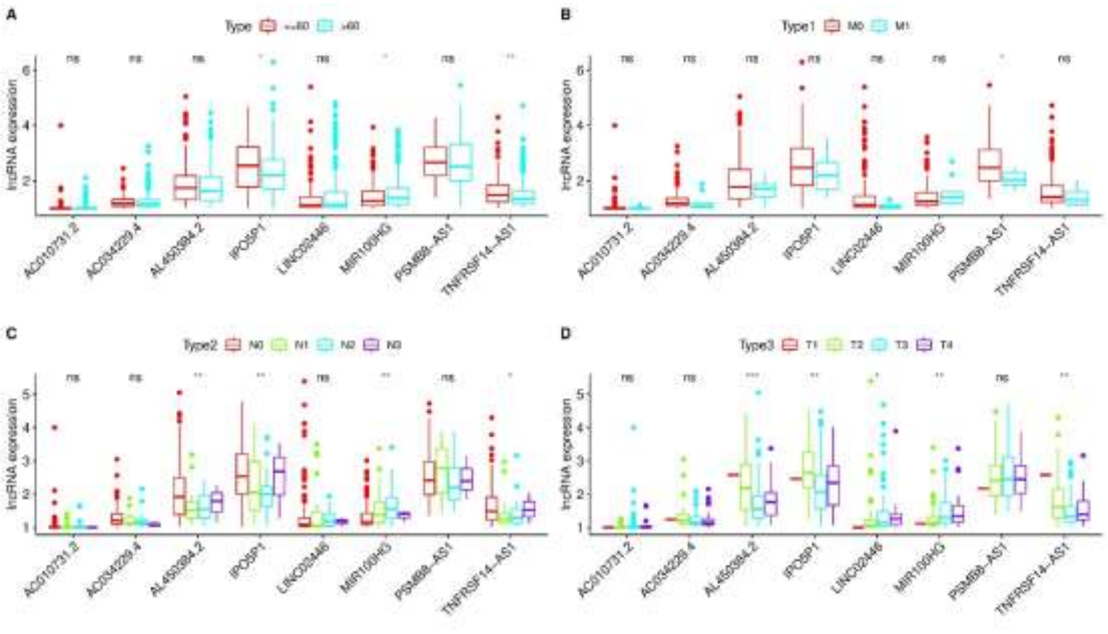

Figure S2: Expression of 8 pyroptosis-related lncRNAs and their clinical characteristics. (A) gender, (B) M-stage, (C) N-stage, (D) T-stage.

Table S1. Univariate analysis of the pyroptosis-related lncRNAs cohort

lncRNA	HR	HR.95L	HR.95H	pvalue
ZNF460-AS1	0.363784	0.186894	0.708096	0.002923
AC008760.1	0.55144	0.375899	0.808958	0.002332
AC002128.1	0.437272	0.238882	0.800426	0.007326
AC104825.1	0.613692	0.473013	0.796211	0.000237
AC009065.8	0.441701	0.279361	0.698379	0.000473
AC006042.1	0.735753	0.593684	0.91182	0.005059
AL157402.2	1.91486	1.285488	2.852372	0.001398
AC008764.6	0.358454	0.172082	0.746674	0.00614
AC018809.1	0.397489	0.228904	0.690233	0.001051
AL109811.3	0.65926	0.486379	0.893592	0.007253
AC104532.2	0.435053	0.247589	0.764455	0.003805
AC006942.1	0.490517	0.286279	0.840462	0.009526
LUCAT1	1.952342	1.34502	2.83389	0.000433
SH3BP5-AS1	0.516136	0.358874	0.742312	0.000361
AL121895.2	0.581616	0.390994	0.86517	0.007478
RPARP-AS1	0.517924	0.345613	0.776145	0.001433
AL031651.2	2.611781	1.541846	4.424179	0.000357
AC009120.2	0.449654	0.287841	0.70243	0.000445
ZNF213-AS1	0.570726	0.388196	0.839082	0.004342
AC005387.1	0.380801	0.212257	0.683179	0.001205
AL355472.1	0.65006	0.47357	0.892324	0.007702
AL139123.1	0.386823	0.195842	0.764044	0.006239
AL031714.1	0.481427	0.283341	0.817994	0.006877
RAD51-AS1	0.643774	0.463726	0.893728	0.008508
PTPRJ-AS1	1.939509	1.253545	3.000848	0.002932
AC004148.2	0.554619	0.403452	0.762424	0.000283
LYST-AS1	2.302676	1.225525	4.326565	0.009543
AL355488.1	0.63764	0.459337	0.885155	0.007168
PTOV1-AS2	0.562532	0.423576	0.747074	7.06E-05
PSMA3-AS1	0.554611	0.380273	0.808875	0.002202
SAMD12-AS1	0.39816	0.209855	0.755431	0.004828
AC009065.4	0.497734	0.314757	0.78708	0.002845
LINC01089	0.662408	0.504048	0.87052	0.00313
AC099343.2	0.401022	0.215347	0.746787	0.003972
IPO5P1	0.662639	0.547056	0.802643	2.58E-05
AC021321.1	0.193433	0.083245	0.449475	0.000134
AC010618.2	0.386414	0.194249	0.768683	0.006735
AC034229.4	0.273448	0.129061	0.57937	0.000712
LINC00115	0.361574	0.175707	0.744057	0.005729
AC011468.1	0.499466	0.353323	0.706059	8.47E-05
AL133410.1	0.355502	0.205947	0.61366	0.000205
ZKSCAN2-DT	0.481976	0.282013	0.823723	0.007605

AC005785.1	0.405465	0.221532	0.742112	0.003422
AL513477.2	0.410105	0.222627	0.755463	0.004241
AL596223.1	1.742638	1.191072	2.549627	0.004229
TMEM51-AS1	0.555595	0.392925	0.785612	0.000884
ZNF32-AS2	0.468806	0.277145	0.79301	0.004732
AL645940.1	0.426043	0.228083	0.795818	0.007443
AC005840.4	0.422997	0.269215	0.664623	0.00019
SNHG20	0.532355	0.356514	0.794924	0.002057
AC010326.3	0.635449	0.483383	0.835354	0.001158
AL162258.2	0.360162	0.188943	0.686537	0.001918
LINC01871	0.803151	0.6814	0.946656	0.008961
ETV5-AS1	2.167217	1.266142	3.709559	0.004795
AC131210.1	2.166911	1.203887	3.900286	0.009916
AC073335.2	0.610162	0.466837	0.797491	0.000299
LINC01355	0.610979	0.422849	0.88281	0.008697
STAG3L5P-PVRIG2P-PILRB	0.255515	0.13433	0.486029	3.19E-05
AC063948.1	0.508729	0.308392	0.83921	0.008136
ARHGAP27P1-BPTFP1-KPNA2P3	0.634395	0.468182	0.859616	0.003327
RBMS3-AS3	2.316836	1.518084	3.535856	9.81E-05
AL353622.1	0.445416	0.287652	0.689707	0.000289
AC011477.3	0.672853	0.513386	0.881855	0.004092
AC003102.1	0.6702	0.499335	0.899531	0.007696
AL022328.2	0.555717	0.383928	0.804373	0.001847
AC022306.2	0.461552	0.267042	0.797742	0.005617
AC089983.1	1.905992	1.198623	3.030814	0.00642
TGFB2-AS1	1.65711	1.131102	2.427731	0.009535
AL591895.1	0.744675	0.622041	0.891485	0.001322
AL022322.1	0.559295	0.406384	0.769741	0.000363
LINC02446	0.706336	0.557567	0.8948	0.003963
AC093726.2	0.40518	0.206782	0.79393	0.00848
AC034236.2	0.34261	0.189799	0.61845	0.000379
U47924.1	0.358431	0.174581	0.735891	0.005181
AL390728.6	0.591615	0.467909	0.748027	1.16E-05
USP30-AS1	0.700927	0.553726	0.88726	0.003132
AL035563.1	0.453435	0.25572	0.804015	0.006801
AC234772.2	1.605536	1.142722	2.255795	0.006354
AC068620.2	0.373497	0.181085	0.770355	0.007669
AC104971.2	2.310204	1.266398	4.214352	0.006334
AC093788.1	0.405874	0.236773	0.695745	0.001041
AC135050.3	0.389924	0.206042	0.737911	0.003806
AC010201.2	0.380137	0.182416	0.792167	0.009826
AC105177.1	2.520811	1.534613	4.140776	0.000261
AC120053.1	0.545002	0.391278	0.759122	0.000331

AC010542.5	0.551084	0.399441	0.760296	0.000285
AC025171.4	0.456601	0.255569	0.815767	0.008104
AC243830.2	0.416478	0.21736	0.798001	0.008289
AC087741.1	0.507989	0.349847	0.737617	0.000372
AC010731.2	2.733902	1.767253	4.229285	6.24E-06
MIR100HG	1.366534	1.084273	1.722275	0.00816
AC078880.5	0.324983	0.151189	0.698556	0.003992
AC084871.1	1.929952	1.227348	3.034765	0.004413
AC020663.2	0.441619	0.240812	0.809872	0.008253
AC016737.1	0.342604	0.16454	0.713368	0.004202
AF178030.1	2.018385	1.186306	3.434086	0.009597
AL355102.1	1.871683	1.230276	2.84749	0.003412
SNHG16	1.572311	1.11788	2.211474	0.009316
AL139089.1	0.44871	0.278078	0.724044	0.001028
TNFRSF14-AS1	0.374913	0.243482	0.57729	8.40E-06
AL136295.2	0.230709	0.108925	0.488655	0.000128
AC005726.3	0.623718	0.46575	0.835263	0.001535
AC027020.2	0.371091	0.217408	0.63341	0.000279
AC115989.1	1.882924	1.179253	3.006481	0.008036
AC104051.2	1.947026	1.266024	2.994343	0.002413
AC007038.2	0.538299	0.343342	0.843958	0.006947
AL354919.2	0.6465	0.512065	0.816229	0.000245
AL450384.2	0.53688	0.403538	0.714282	1.96E-05
AC078880.3	0.545008	0.371788	0.798931	0.001869
PSMB8-AS1	0.764962	0.631948	0.925973	0.005976
AC245884.8	0.52928	0.336685	0.832045	0.00584
AL021707.6	0.603261	0.464769	0.783021	0.000146
AL390719.2	0.790673	0.68751	0.909315	0.000992
AC055822.1	0.544219	0.377798	0.78395	0.001087
AC024060.1	0.569668	0.443987	0.730926	9.66E-06
AC008610.1	0.562016	0.402892	0.783987	0.000691
AC011477.2	0.653545	0.482586	0.885067	0.005977
ZNF32-AS1	0.431919	0.238102	0.783507	0.005729
AL355353.1	0.691152	0.575104	0.830616	8.19E-05

Table S2. LASSO regression coefficients of sixteen pyroptosis-related lncRNAs.

lncRNA	Coefficient
AL031651.2	0.144994
IPO5P1	-0.18597
AC021321.1	-0.43346
AC034229.4	-0.37206
AC011468.1	-0.28031
STAG3L5P-PVRIG2P-PILRB	-0.39037
RBMS3-AS3	0.259661
LINC02446	-0.44938
USP30-AS1	-0.17052
AC010731.2	1.395285
MIR100HG	0.064872
TNFRSF14-AS1	-0.51875
AL354919.2	-0.12758
AL450384.2	-0.28801
PSMB8-AS1	-0.0486
AL355353.1	-0.01124

Table S3: Proportional Hazards Assumption of nomogram Cox Regression

	chisq	df	P
age	0.00436	1	0.95
Gender	0.50937	1	0.48
T stage	0.51808	1	0.47
N stage	1.91813	1	0.17
M stage	0.02906	1	0.86
Stage	1.92675	1	0.17
Risk score	2.32676	1	0.13
GLOBAL	8.07666	7	0.33

Table S4. Gene set enrichment GO analysis results of high risk group 8 pyroptosis-related lncRNAs (Top 10 p-value)

NAME	SIZE	NES	NOM p-val	FDR q-val	LEADING EDGE
GOMF_PROTEIN_TRANSPORTER_ACTIVITY	29	1.73256	0.001908	1	tags=48%, list=12%, signal=55%
GOMF_OLIGOSACCHARIDE_BINDING	16	1.705475	0.00202	0.991089	tags=63%, list=11%, signal=70%
GOMF_MONOSACCHARIDE_BINDING	67	1.734055	0	1	tags=54%, list=13%, signal=62%
GOMF_MANNOSE_BINDING	19	1.771577	0	1	tags=68%, list=16%, signal=81%
GOMF_DISULFIDE_OXIDOREDUCTASE_ACTIVITY	40	1.756601	0	1	tags=70%, list=16%, signal=84%
GOMF_2_IRON_2_SULFUR_CLUSTER_BINDING	22	1.729062	0.005758	0.91257	tags=50%, list=10%, signal=55%
GOCC_AZUROPHIL_GRANULE_LUMEN	91	1.740597	0.004107	1	tags=63%, list=17%, signal=76%
GOCC_AZUROPHIL_GRANULE	153	1.685629	0.012474	1	tags=55%, list=15%, signal=65%
GOBP_PEPTIDYL_PROLINE_MODIFICATION	58	1.69061	0.004024	1	tags=45%, list=14%, signal=52%
GOBP_CHAPERONE_MEDIATED_PROTEIN_FOLDING	63	1.7086	0	1	tags=48%, list=11%, signal=54%

Table S5. Top 10 function of 8 pyroptosis-related lncRNAs from GO enrichment analysis results of low-risk group (Top 10 p-value)

NAME	SIZE	NES	NOM p-val	FDR q-val	LEADING EDGE
GOMF_LIGAND_ACTIVATED_TRANSCRIPTION_FACTOR_ACTIVITY	52	-1.77725	0	0.93056	tags=52%, list=14%, signal=61%
GOBP_ORGANISM_EMERGENCE_FROM_PROTECTIVE_STRUCTURE	22	-1.7729	0	0.506387	tags=59%, list=16%, signal=70%
GOBP_NEGATIVE_REGULATION_OF_CHOLESTEROL_EFFLUX	15	-1.69327	0.005952	1	tags=53%, list=16%, signal=63%
GOBP_PHOSPHATIDYLCHOLINE_ACYL_CHAIN_REMODELING	29	-1.65638	0	1	tags=45%, list=10%, signal=50%
GOBP_BRANCHING_INVOLVED_IN_MAMMARY_GLAND_DUCT_MORPHOGENESIS	20	-1.63565	0.008065	1	tags=65%, list=18%, signal=79%
GOBP_RETINOIC_ACID_RECEPTOR_SIGNALING_PATHWAY	32	-1.60752	0.005859	1	tags=50%, list=17%, signal=60%
GOBP_NEGATIVE_REGULATION_OF_STEROL_TRANSPORT	23	-1.60312	0.021526	1	tags=52%, list=16%, signal=62%
GOBP_DIACYLGLYCEROL_METABOLIC_PROCESS	25	-1.6001	0.006036	1	tags=48%, list=14%, signal=56%
GOBP_HISTONE_H3_K4_TRIMETHYLATION	17	-1.59473	0.009728	1	tags=76%, list=16%, signal=90%
GOBP_PHOSPHATIDYLETHANOLAMINE_ACYL_CHAIN_REMODELING	23	-1.58862	0.014199	1	tags=43%, list=10%, signal=48%

Table S6. KEGG pathway analysis results 8 pyroptosis-related lncRNAs in high-risk group (Top 10 p-value).

NAME	SIZE	NES	NOM.pvalue	FDR	LEADING EDGE
KEGG_PROTEASOME	46	1.6171805	0.028056113	0.8888676	tags=76%, list=13%, signal=88%
KEGG_PRION_DISEASES	35	1.6146219	0.011976048	0.45655707	tags=54%, list=16%, signal=65%
KEGG_GLYCOSAMINOGLYCAN_BIOSYNTHESIS_CHONDROITIN_SULFATE	22	1.5632548	0.029239766	0.5445688	tags=64%, list=16%, signal=75%
KEGG_PURINE_METABOLISM	156	1.5081213	0.022633744	0.7085648	tags=55%, list=24%, signal=72%
KEGG_AMINO_SUGAR_AND_NUCLEOTIDE_SUGAR_METABOLISM	42	1.5065918	0.036144577	0.5736164	tags=50%, list=16%, signal=59%
KEGG_RENIN_ANGIOTENSIN_SYSTEM	17	1.4977536	0.03426124	0.5124177	tags=59%, list=15%, signal=69%
KEGG_PROTEIN_EXPORT	24	1.4931266	0.042944785	0.45845512	tags=54%, list=17%, signal=65%
KEGG_CYTOKINE_CYTOKINE_RECEPTOR_INTERACTION	264	1.476344	0.049603175	0.4640261	tags=45%, list=15%, signal=53%
KEGG_AUTOIMMUNE_THYROID_DISEASE	50	1.4361321	0.114173226	0.57255715	tags=40%, list=10%, signal=45%
KEGG_SYSTEMIC_LUPUS_ERYTHEMATOSUS	55	1.4357249	0.09325397	0.51699173	tags=65%, list=18%, signal=80%

Table S7. KEGG pathway analysis results 8 pyroptosis-related lncRNAs in low-risk group (Top 10 p-value).

NAME	SIZE	NES	NOM.pvalue	FDR	LEADING EDGE
KEGG_LINOLEIC_ACID_METABOLISM	29	-1.59555	0.00996	0.558545	tags=48%, list=13%, signal=55%
KEGG_ALPHA_LINOLENIC_ACID_METABOLISM	19	-1.43757	0.053254	1	tags=53%, list=12%, signal=60%
KEGG_PRIMARY_BILE_ACID_BIOSYNTHESIS	16	-1.3933	0.079612	1	tags=38%, list=5%, signal=40%
KEGG_DRUG_METABOLISM_CYTOCHROME_P450	71	-1.36543	0.054264	1	tags=41%, list=15%, signal=48%
KEGG_METABOLISM_OF_XENOBIOTICS_BY_CYTOCHROME_P450	69	-1.34506	0.068762	0.90958	tags=41%, list=15%, signal=48%
KEGG_GLYCEROPHOSPHOLIPID_METABOLISM	77	-1.34308	0.035573	0.767071	tags=34%, list=15%, signal=40%
KEGG_RETINOL_METABOLISM	64	-1.32524	0.048544	0.732856	tags=38%, list=13%, signal=43%
KEGG_NOTCH_SIGNALING_PATHWAY	47	-1.32319	0.084314	0.649976	tags=64%, list=25%, signal=85%
KEGG_ADIPOCYTOKINE_SIGNALING_PATHWAY	67	-1.27198	0.109756	0.783415	tags=46%, list=18%, signal=56%
KEGG_DORSO_VENTRAL_AXIS_FORMATION	24	-1.21072	0.184	0.966428	tags=42%, list=14%, signal=48%
